# High honeybee abundances reduce wild bee abundances on flowers in the city of Munich

**DOI:** 10.1007/s00442-021-04862-6

**Published:** 2021-02-07

**Authors:** Susanne S. Renner, Marie Sophie Graf, Zoe Hentschel, Helen Krause, Andreas Fleischmann

**Affiliations:** 1 Systematic Botany and Mycology, University of Munich (LMU), Menzinger Str. 67, 80638 Munich, Germany; 2 Botanische Staatssammlung München, Menzinger Str. 67, 80638 Munich, Germany

**Keywords:** Exploitative competition, Honeybees, Wild bees, Resource consumption, Urban bee keeping

## Abstract

The increase in managed honeybees (*Apis mellifera*) in many European cities has unknown effects on the densities of wild bees through competition. To investigate this, we monitored honeybees and non-honeybees from 01 April to 31 July 2019 and 2020 at 29 species of plants representing diverse taxonomic and floral-functional types in a large urban garden in the city of Munich in which the same plant species were cultivated in both years. No bee hives were present in the focal garden, and all bee hives in the adjacent area were closely monitored by interviewing the relevant bee keepers in both 2019 and 2020. Honeybee numbers were similar in April of both years, but increased from May to July 2020 compared to 2019. The higher densities correlated with a significant increase in shifts from wild bee to honeybee visits in May/June/July, while visitor spectra in April 2019 and 2020 remained the same. Most of the species that experienced a shift to honeybee visits in 2020 were visited mostly or exclusively for their nectar. There were no shifts towards increased wild bee visits in any species. These results from a flower-rich garden have implications for the discussion of whether urban bee keeping might negatively impact wild bees. We found clear support that high honeybee densities result in exploitative competition at numerous types of flowers.

## Introduction

It is notoriously difficult to provide unambiguous evidence of competition, particularly in mobile organisms (Goulson 2003). Because of this there is no clear agreement whether increased honeybee densities have a negative impact on wild bee diversity or abundance via exploitative competition for nectar and pollen (Gunnarsson and Federsel 2014; Lindström et al. 2016; Geslin 2017; Mallinger et al. 2017; Wojcik et al. 2018). Most studies so far have focused on agricultural settings to address the question of resource overlap and competition between honeybees and wild bees. In Central Europe, however, cities are now a refuge for several species of wild bees (Sirohi et al. 2015; Banaszak-Cibicka et al. 2018; Hofmann et al. 2019), and some have higher bee diversities than similarly-sized arable areas or forest, probably because of high plant diversity, longer-lasting flowering season, and near-absence of pesticides and herbicides.

The new role of cities as refugia for wild bees raises the question whether the current increase in urban honeybee keeping (Lorenz and Stark 2015) might negatively impact wild bees in cities by depleting their nectar and/or pollen resources. The question is difficult to answer, because the European dark honeybee (*Apis mellifera mellifera*) is a native European species that has coexisted with European wild bee species for thousands of years (Dams 1978) during which time both groups simultaneously had to cope with numerous changes in flower abundances and local climate. To detect significant ongoing changes in foraging competition between honeybees and wild bees, data are required from settings in which the abundances of honeybees change, but those of floral resources and wild bee nesting sites do not.

Here we report such data from two flowering seasons in a botanical garden in an urban setting in which it was possible to monitor wild bee and honeybee visits in a wide range of plant species. The plants were studied at the same locations and with the same methods in both years (during short intervals distributed over numerous sunny days for a total of about 9 h/species), and honeybee numbers were estimated by monitoring all hives in the surrounding area and interviewing their owners. The expectation was that under food competition, increased honeybee densities at a particular flower species would shift the relative proportions of wild bees at that plant and time. The bee-rich garden in which our study was conducted contains no bee hives, so that all foraging honeybees come from the surrounding area (Hofmann et al. 2018; Hofmann and Renner 2020). This experimental set-up captures the situation in many European cities in which bees from hives on roofs and balconies forage in near-by parks, private gardens, or allotment gardens (Beckedorf 2015; Hofmann and Renner 2018; Wojcik et al. 2018). Given the lack of data on the effects of urban bee keeping on wild bees (Geslin 2017; Wojcik et al. 2018), we designed this study to help inform conservation and management measures in cities.

## Materials and methods

The study took place in the Munich Botanic Garden from 01 April to 31 July 2019 and 2020. The garden opened in May 1914, covers about 21 hectares and borders on the 210-hectar-large Nymphenburg Palace Park at 48°09′45″ N and 11°30′06″ E, at 500 m above sea level. It is currently home to 106 bee species (including honeybee) whose abundances were scored in 2015–2017 by repeated monitoring walks (Hofmann et al. 2018). Several cavity nest boxes for solitary bees are located in the garden, but no honeybee hives have ever been placed there. The botanical garden provides a flower-rich habitat with thousands of native and cultivated species and varieties in flower beds and near-natural meadows throughout the year. Its layout of paths and beds is protected as a cultural monument, and all beds are watered and cared for by 44 gardeners, whose professional task and goal is to maintain a beautiful display of healthy plants all year long. Since 1795, 324 species of bees have been recorded from Munich (Hofmann and Renner 2020) and 123 from the Botanical Garden from 1997–2017 (79 species in 1997–1999, 106 in 2015–2017, with an overlap of 62 species; Bembé et al. 2001; Hofmann et al. 2018).

From 01 April until 31 July 2019 and 2020, we counted bees that alighted and foraged on the flowers of 14 species in April and May, and 15 species in June and July, one plant species (*Nepeta mussinii*) was observed in both April and May. Plants were observed at the same sites in both years and had the same distances to the surrounding honeybee hives in both years. Bees were counted during many 5-min intervals on 15–50 flowers or inflorescences for a total of about 9 h per species, with the number of flowers chosen so that all bees could be seen and counted with precision. Observations were only made during dry, sunny or at most slightly overcast days. Herbarium vouchers were made of each species and deposited in the Munich herbarium.

In both years, all four bee keepers in the Nymphenburg Palace park (S. Fritz, M. Högner, A. Kromer, and Mr. Kostrow) were interviewed about the health and size of their bee hives.

## Results

**Fig. 1:**
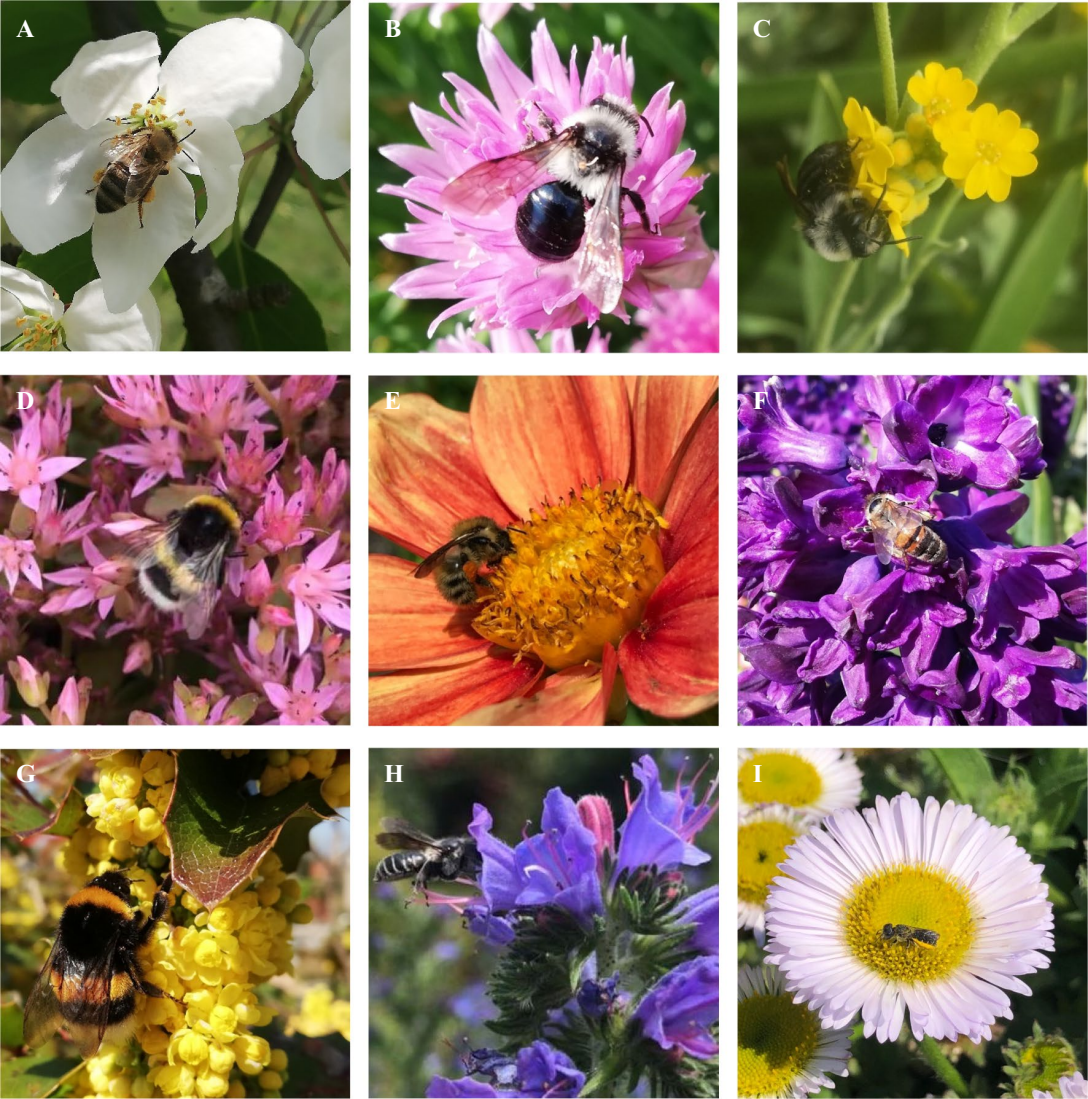
Examples of plant and bee species monitored in this study. **a** *Apis mellifera* on *Malus baccata*, **b** *Andrena cineraria* on *Allium schoenoprasum*, **c** *Andrena cineraria* on *Aurinia saxatilis*, **d** *Bombus lucorum *s.l*.* on *Phedimus spurius*, **e** *Bombus pascuorum* on *Dahlia* hybrid ‘Fee’, **f** *Apis mellifera* on *Hyacinthus orientalis* hybrid, **g** *Bombus terrestris* on *Mahonia aquifolium*, **h** *Hoplitis adunca* on *Echium vulgare* , **i** *Heriades truncorum* on *Erigeron glaucus*

**Fig. 2:**
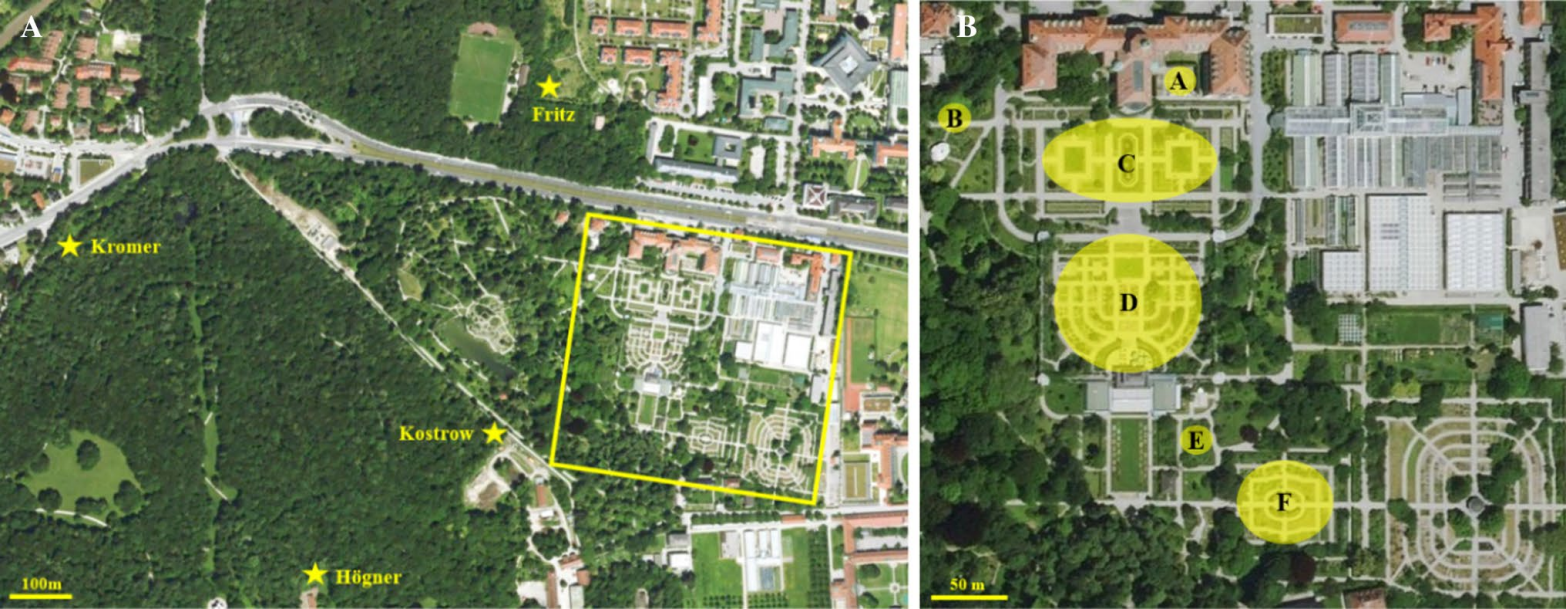
Locations of bee hives and plant species monitored in this study. **a** Locations of the hives of the beekeepers Fritz, Kromer, Kostrow, and Högner. The Botanical Garden Munich is shown in the yellow square. In **b** the yellow square is enlarged. The letters A–F stand for the locations of the 29 plant species numbers in Table 1 numbered continuously. A: plant no. 25; B: plant no. 14; C: plant nos. 2, 5, 6, 10, 11, 16, 22, 24, 26 and 28; D: plant nos. 3, 4, 7, 9, 12, 17, 18, 19, 20, 21, 23, 27 and 29; E: plant no. 8; and F: plant nos. 1, 13 and 15

In total, we observed 9.328 honeybees and 6.460 wild bees over 172 h in 2019 and 18.630 honeybees and 6.281 wild bees over 264 h at the same 29 plant species in 2020 (Table 1). The focal plants represented different taxonomic and floral-functional types (Fig. 1), including native species and horticultural forms, species adapted to bee pollination (e.g., the Lamiaceae *Lavandula angustifolia*, *Leonurus cardiaca*, *Stachys byzantina*; Asteraceae such as *Taraxacum*) as well as species pollinated by other insects, such as flies and butterflies, in their native habitats (e.g., *Hyacinthus*) or species in areas naturally devoid of honeybees (e.g., New World *Dahlia*, *Echinacea*, and *Mahonia aquifolium*). The species and densities of other flowering plants (not monitored for this study) present in the botanical garden in both years were similar. Honeybees were observed at all plant species (Table 1) and at all distances from the hives (Fig. 2). The resource overlap within the habitat, i.e., the percentage of plant species used by both honeybees and wild bees was almost complete, suggesting food competition. Two species, *Helianthemum* and *Cotoneaster*, in 2019 were visited by both wild bees and honeybees, but in 2020 only by honeybees (Table 1).

**Table 1:** Plant species studied in spring and summer of 2019 and 2020 with their geographic origin and number of honeybees and wild bees per hour, and whether bees foraged for pollen and/or nectar

Plant species and hours of observations and month (2019/2020)	Geographic origin	Honeybees/h		Wild bees/h		Nectar/pollen		Shift
		2019	2020	2019	2020	2019	2020	
April and May								
* Allium schoenoprasum *L. (6/10, April)	Eurasia & North America	50	51	1	4	Pollen and nectar	Pollen and nectar	
*Aurinia saxatilis* (L.) Desv. (6/9, April)	Central Europe, Southern Europe, Asia Minor	2	1	32	15	Pollen and nectar	Pollen and nectar	
*Cotoneaster horizontalis* Decne. (6/10, May)	Western China, Taiwan	103	124	2	0	Pollen	Pollen	(X)
*Erysimum allionii* Kuntze ‘Orange’ (6/10, May)	Greece	64	52	5	6	Pollen and nectar	Pollen and nectar	
*Euonymus alatus* (Thunb.) Siebold (4/9, April)	Japan, Central China	31	23	5	2	Nectar	Nectar	
*Hyacinthus orientalis* L. hybrid (5/10, April)	Asia Minor, Middle East and SW Asia	52	35	2	5	Pollen and nectar	Pollen and nectar	
*Mahonia aquifolium* (Pursh) Nutt. (6/10, April)	Pacific America	37	29	7	5	Pollen and nectar	Pollen and nectar	
*Malus baccata* (L.) Borkh. var. *mandshurica *(6/10, April)	Central Japan, Central China, Korea	47	38	15	2	Pollen and nectar	Pollen and nectar	
*Muscari botryoides* Mill. (8/9, April)	Central and Southern Europe, Asia	8	25	7	8	Pollen and nectar	Pollen and nectar	
*Narcissus pseudonarcissus* L. (6/1, April)	Western Europe	4	1	14	7	Pollen and nectar	Pollen	
*Nepeta mussinii* Spreng. ex Henck. ‘Superba’ (6/10, April)	Eastern Turkey, Northwest Iran	7	8	35	26	Pollen and nectar	Pollen and nectar	
*Nepeta mussinii *(6/10, May)	Ditto	76	84	7	53	Pollen and nectar	Pollen and nectar	
* Rhododendron* ‘Roselyn’ (7/10, May)	Himalayas	40	12	52	49	Pollen	Pollen	
*Rubus fruticosus* L. s.l (7/10, May)	Europe, North Africa, Asia, North America	78	113	15	11	Pollen and nectar	Pollen and nectar	(X)
*Taraxacum* sect*. Taraxacum *(7/10, April)	Central Europe	28	26	14	5	Pollen and nectar	Pollen and nectar	
June and July								
*Brassica juncea* (L.) Czern. (4/8, June)	Western and Central Asia	73	39	23	21	Pollen and nectar	Pollen and nectar	
*Dahlia* hybrid ‘Fee’ (7/10, July)	Mexico and Central America	62	63	75	37	Pollen and nectar	Pollen and nectar	
*Echinacea purpurea* (L.) Moench (7/10, July)	North America	60	55	37	12	Nectar	Nectar	
*Echinops banaticus* Rochel & Borza (11/10, July)	Southeast Europe	72	169	48	136	Nectar	Nectar	
*Echium vulgare* L. (7/10, June/July)	Europe, Western Asia	55	101	49	46	Nectar	Nectar	
*Erigeron glaucus* KerGawl. (8/10, June)	North America	7	56	90	84	Pollen	Pollen	
*Helianthemum* hybrid (1/8, June)	Central Europe, Mediterranean	169	91	6	0	Pollen (flowershave no netcar)	Pollen (flowers have no nectar)	
*Heterotheca villosa* (Pursh) Shinners (8/10, June)	Mexico	23	134	51	1	Pollen	Pollen	X
*Lavandula angustifolia* Mill. (7/9, July)	Mediterranean	74	132	51	22	Nectar	Nectar	X
*Leonurus cardiaca* L. (7/10, July)	Central Asia, Southeast Europe	53	67	43	8	Nectar	Nectar	X
*Reseda alba* L. (7/5, June)	Europe, Asia, North Africa	26	49	100	18	Nectar	Nectar	X
*Salvia farinacea* Benth. (5/10, June/July)	Mexico, S USA	9	23	41	6	Nectar	Nectar	X
*Phedimus spurius* (M.Bieb.) ‘t Hart (6/10, July)	North America, Asia	179	231	53	21	Nectar	Nectar	X
*Stachys byzantina* K.Koch (8/10, June/July)	Asia Minor, Caucasus	70	87	33	11	Nectar	Nectar	X
*Symphytum officinale* L. (2/10, June)	Eurasia, Spain, China	29	17	51	42	Nectar	Nectar	

Also shown are resource use and if there is a shift in the proportions of honeybees to wild bees between 2019 and 2020, with weak shifts indicated by (X) and strong shifts by X

Twelve (41%) of the 29 species were visited as pollen and nectar sources; five (17%) were only pollen sources; and 11 (38%) were only nectar sources. The only plant used differently in the two study years was *Narcissus pseudonarcissus*, which in 2019 was visited for both pollen and nectar, but in 2020 only for pollen. Two of the nine species (22%) that experienced a shift in their visitor spectra in 2020 compared to 2019 were pollen-only sources, six (67%) were nectar-only sources, and one (11%) was exploited for both its pollen and nectar, implying that seven (78%) of the species that experienced a shift to fewer wild bees in 2020 were visited for their nectar.

In April 2019 and 2020, honeybee densities remained identical (Table 2), but they increased from May to July 2020 compared to 2019 (Table 2). This increase correlated with significantly fewer wild bee visits in nine of the 20 May/June/July-flowering species, while visitor spectra did not change in the ten April-flowering species (Table 1, which shows 30 observations, because *N. mussinii* was observed in April and May; χ^2^ = 6.43, *df* = 1, *P* = 0.05). All observed shifts in visitor spectra were in the direction of increased honeybee numbers (Table 1).

**Table 2:** Honeybee hives near the botanical garden Munich in 2019 and 2020 (see Fig. 2 for the location of hives)

Month	Bee hives 2019	Honey bees 2019 (millions)	Bee hives 2020	Honeybees 2020 (millions)
April	≤ 33	≤ 0.7–1.3	33	0.7–1.3
May	≤ 33	≤ 0.7–1.3	41	1.0–1.6
June	33	0.7–1.3	44/45	1.3–1.8
July	33	0.7–1.3	44	1.3–1.9

The numbers of bees per hive were estimated by the four bee keepers who owned the hives (“Materials and methods”). The increase in June 2020 is due to natural reproduction (swarming of bees)

## Discussion

Despite the large diversity and abundance of flowers available at our study site, a 21-hectar-large botanical garden, we found a significant negative relationship between the densities of honeybees and those of flower-visiting wild bees, almost regardless of flower type (Fig. 1; Tables 1, 2). That the higher resource depletion by foraging honeybees in May, June, and July 2020 compared to 2019 negatively affected the abundances of foraging wild bees, matches evidence that the experimental addition of honeybee colonies negatively impacts bumblebees that overlap with honeybees in resource use (Wojcik et al. 2018). Per year, a honeybee colony harvests 10–60 kg of pollen and 20–150 kg of honey, which translates to 5–9000 kg pollen and 10–22.500 kg honey/km^2^/year (Goulson 2003). These numbers suggest that honeybees must use a substantial proportion of floral resources at any one time and place, and as our data show (Table 1), food competition occurred not only at flowers providing both nectar (sugars) and pollen (protein) but also at flowers that provide only pollen. Bees are often more taxonomically restricted in their pollen collection than in their nectar collection (Cane and Sipes 2006); however, only 23% of 445 wild bees that occur in Germany (and for which data on pollen preferences are available) are pollen specialists (Hofmann et al. 2019). Most European wild bees are also much smaller than honeybees and have short average flight distances (Hofmann et al. 2020), which further decreases their ability to avoid competition by foraging at more distant plant populations.

Although our study demonstrates the depressing effects of increased honeybee densities on the simultaneous proportions of wild bees at flowers of the same species, we lack data on the fitness effects of this observation. It is plausible that in the summer of 2020, wild bees had to travel further and/or use less profitable flowers compared to 2019, but to determine whether this had non-trivial effects on their fitness would require competitive exclusion experiments combined with longer-term studies of wild bee populations. To our knowledge, no such study has been carried out (Steffan-Dewenter and Tscharntke 2000; Goulson 2003; Wojcik et al. 2018). That the visitor shifts observed in 2020 might instead have been due to lower abundances of wild bee species, or to higher or lower flower densities, seems implausible given the complete consistency of the direction of shifts (from wild bees to honeybees) throughout all three months with higher honeybee densities (Tables 1, 2) and the rich flower diversity and abundance in the botanical garden.

Based on the present results from a resource-rich urban garden, caution should be used when introducing high densities of *Apis mellifera* in cities. The city of Paris in 2018 harboured 7 hives/km^2^, Berlin in 2014 had 6 hives/km^2^, and Hamburg in the same year 5–6 hives/km^2^ (Beckedorf 2015), with an increase in the latter two cities of 125% between 2007 and 2014. In our study area, the densities were 16 hives/km^2^ in 2019 and 22 hives/km^2^ in 2020. In Mediterranean scrubland, densities of 3.5 hives/km^2^ can have measurable negative effects on wild bee communities (Torné-Noguera et al. 2015). Should such high densities persist over longer periods, and should flower densities remain unchanged, strong food competition between honeybees and wild bees is likely and may have negative consequences for the persistence of wild bee populations in cities.
